# Case Report: Abruptio placentae and epileptic seizure after occurrence of perinatal hyperglycaemia in woman with gestational diabetes mellitus and hypertriglyceridemia-induced acute pancreatitis

**DOI:** 10.3389/fendo.2023.1220957

**Published:** 2023-10-18

**Authors:** Yanlang He, Zhijie Huang, Changli Wei, Jianyong Chen

**Affiliations:** ^1^ Medical College of Nanchang University, Nanchang, China; ^2^ Department of Gastroenterology and Hepatology, Jiangxi Provincial People's Hospital, The First Affiliated Hospital of Nanchang Medical College, Nanchang, China

**Keywords:** abruptio placentae, epileptic seizure, hyperglycaemia, hypertriglyceridemia-induced, gestational diabetes mellitus

## Abstract

Hypertriglyceridemia-induced acute pancreatitis seldom occurs in the second trimester of pregnancy with gestational diabetes mellitus. For these patients, the existing knowledge on concomitant hyperglycemia is not sufficient. We report a case of abruptio placentae and epileptic seizure following perinatal hyperglycaemia in woman with gestational diabetes mellitus and hypertriglyceridemia-induced acute pancreatitis. The occurrence of abruptio placentae and epileptic seizure may be associated with concomitant hyperglycemia, and the epileptic seizure was terminated after she underwent treatment with insulin. We should pay more attention to the adverse effects of perinatal hyperglycemia and continue to give appropriate insulin treatment even if patients have passed the acute phase of hypertriglyceridemia-induced acute pancreatitis.

## 1 Introduction

Pregnant women with gestational diabetes mellitus(GDM) usually return to normal blood glucose after delivery due to reduced insulin resistance (1, 2). As a result, the effects and management of perinatal hyperglycemia, especially in women with acute pancreatic disease, have been poorly studied. In addition, hypertriglyceridemia-induced acute pancreatitis (HTG AP) rarely occurs in the second trimester of pregnancy (3–5). Here, we describe a rare case of abruptio placenta and epileptic seizure following perinatal hyperglycaemia in woman with GDM and HTG AP in the second trimester, discuss possible causes, and compare treatment options for concomitant hyperglycaemia in the perinatal period.

## 2 Case description

In October 2022, a 29-year-old multipara with 27 + 2 weeks of amenorrhea was admitted to the emergency department of Jiangxi Provincial People’s Hospital with acute abdominal pain. There was no previous history of gastrointestinal ulcer or pancreatitis. During two previous pregnancies, the patient developed gestational diabetes mellitus. In this pregnancy, she underwent a 75g oral glucose tolerance test at 24 weeks of gestation and found a fasting blood glucose (FBG) level of 8.0mmol/l (>7mmol/l) and a 1-hour postprandial blood glucose level of 10.5mmol/l (>10mmol/l). She was diagnosed with gestational diabetes mellitus according to the latest guideline (6). However, she did not regularly monitor her glucose levels. She has recently been taking in a bit more lipid than usual.

**Figure 1:**
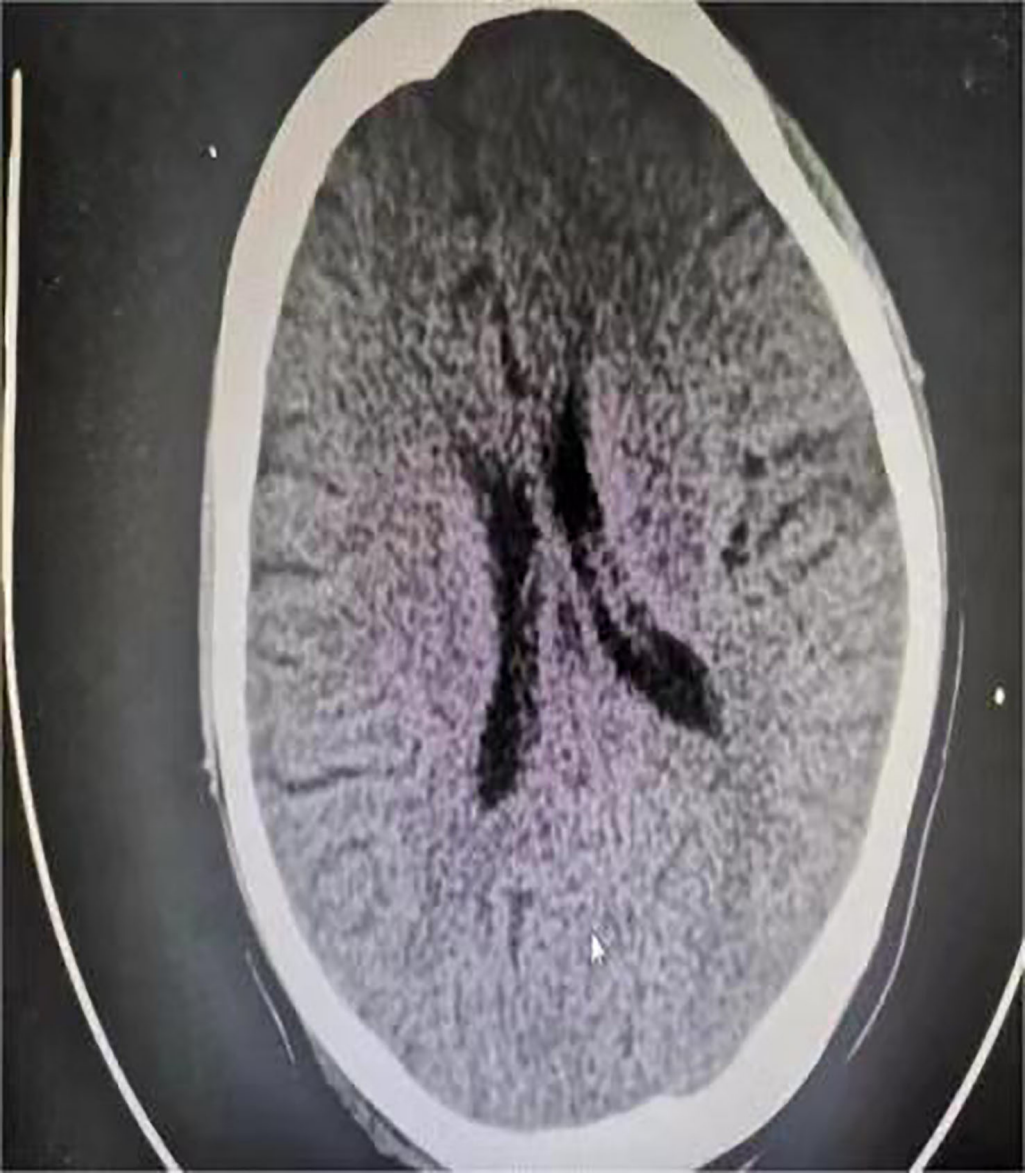
Computed tomography of the patient's head after cesarean section.

**Figure 2:**
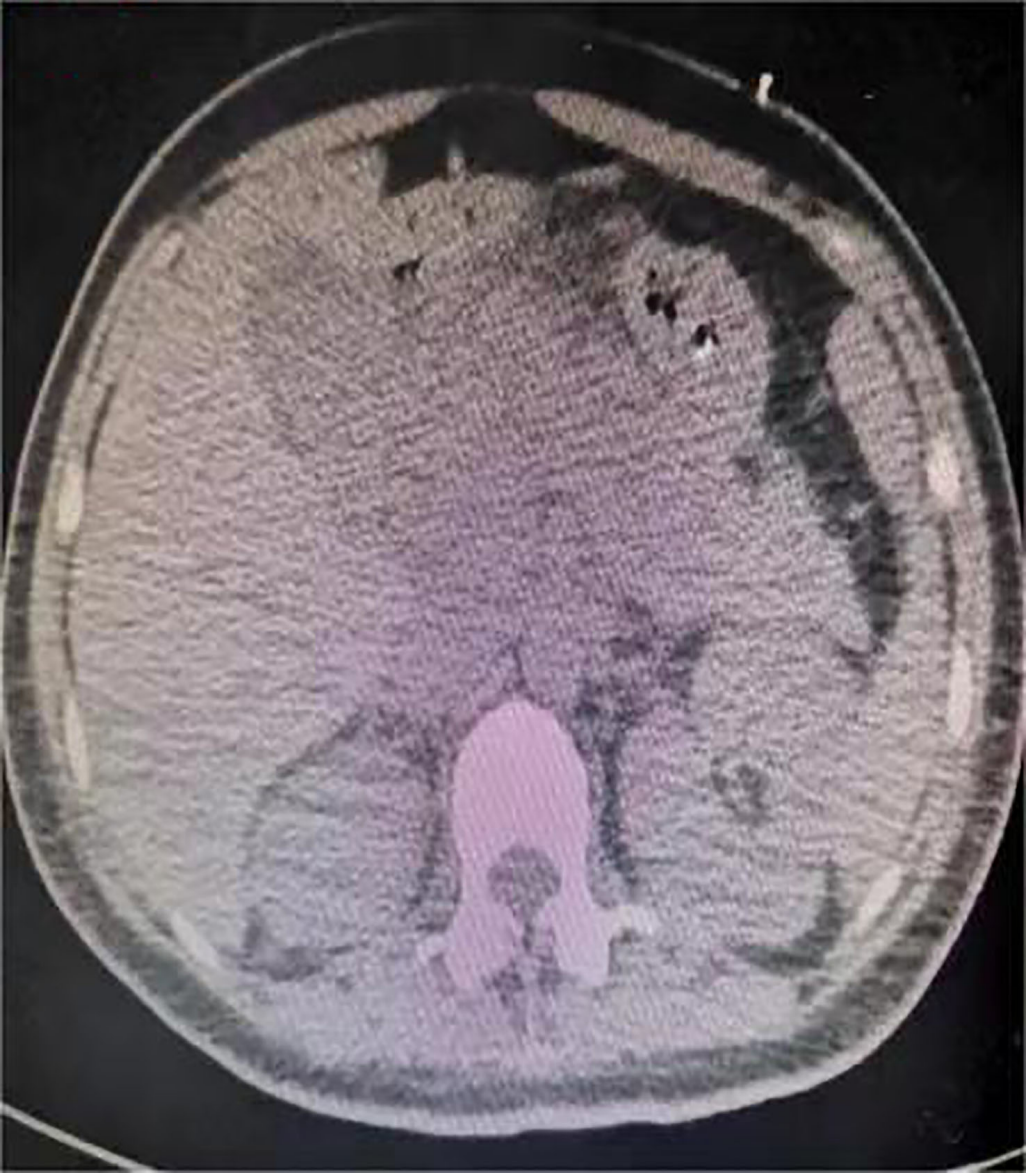
Computed tomography of the patient's abdomen after cesarean section.

**Figure 3:**
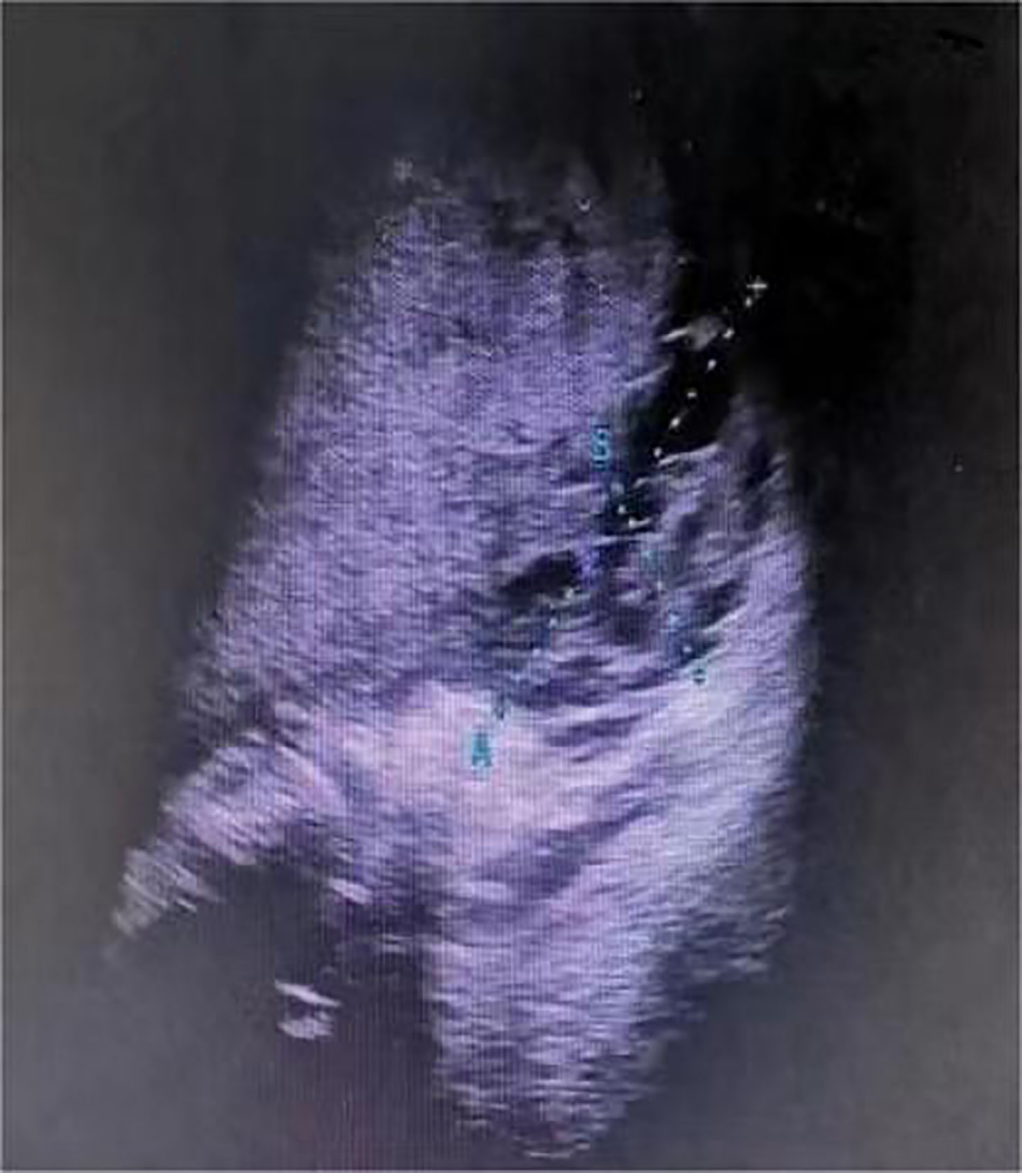
Obstetric ultrasound of the patient before cesarean section.

One day before admission, the patient suddenly developed persistent epigastric pain with nausea and vomiting. Next, she began experiencing pain in her right lower abdomen and vaginal bleeding, and was rushed to the hospital. Upon admission, the patient was in a coma, physical examination: heart rate 126 pulses per minute, blood pressure 123/51mmHg (supported by norepinephrine 0.5ug/kg/min), epileptic seizures, uncooperative nervous system examination. The rest of the physical examination was unremarkable. Her triglyceride was 31.92mmol/L (reference range 0.45 to 1.7mmol/L), amylase 401.2U/L (reference range 35 to 135U/L), white blood cell 20.87*10^9^/L (reference range 4 to 10*10^9^/L), procalcitonin 4.68ng/ml (reference range 0 to 0.05ng/mL), random blood glucose 15.0mmol/L. Blood gas analysis showed pH7.35, lactic acid 0.79mmol/L (reference range 0.5 to 1.7mmol/L), and urine ketone bodies were negative. Computed tomography (CT) of the head (**Figure 1**) was normal, and CT of the abdomen (**Figure 2**) showed that: pancreatic morphology was abnormal and combined with extensive peripheral exudation. Because ultrasound (**Figure 3**) showed mixed echoes posterior to the placenta, abruptio placentae was considered. An emergency Caesarean section was performed on the lower uterine segment to terminate the pregnancy. Unfortunately, the newborn died. After the operation, she started developing epileptic seizures again and transferred to critical care medicine department.

Combined with the typical symptoms of persistent upper abdominal pain, significant increases in blood amylase and triglycerides, imaging findings from abdominal CT, and the exclusion of common causes of acute pancreatitis such as gallstones and alcohol history, we considered acute pancreatitis induced by hypertriglyceridemia as the primary disease. Our treatment measures include active fluid resuscitation, fasting, plasma exchange, nasogastric tube decompression, inhibition of gastric acid secretion and pancreatic enzyme secretion, broad-spectrum antibiotics to prevent infection, nutritional support, analgesia and sedation, invasive ventilator-assisted respiration, traditional Chinese medicine rhubarb to induce diarrhoea, and additional supportive therapies. Given her history of GDM, insulin was continuously infused by micropump and the rate was adjusted based on hourly capillary glucose checks.

After aggressive treatment, patient gradually regained consciousness from the second day, seizures disappeared, blood pressure stabilized, and laboratory tests showed significant reductions in blood glucose, blood lipids, and inflammatory markers. On the fourth to fifth day in the hospital, her condition improved further. On the evening of the fifth day in the hospital, we considered her to have passed the acute phase due to significant improvement in vital signs, inflammatory markers, and imaging findings. She was transferred to the gastroenterology department for continued treatment. However, the patient refused to continue with insulin and was switched to oral metformin 500mg three times daily and acarbose 50mg three times daily to control blood glucose according to the latest guidelines (6).

On the sixth day in the hospital, the patient had a postprandial seizure. She had no history of epilepsy. Temperature, blood pressure and neurological examination were not abnormal.Blood gas analysis showed a pH of 7.37, osmotic pressure of 304mOsm/kgH2O, negative urinary ketone bodies, and normal blood calcium levels. The only positive result was that blood glucose levels exceeded 20 mmol/l during each seizure (**Table 1**), which terminated approximately 5 to 10 minutes after the subcutaneous insulin injection.

**Table 1:** Partial capillary glucose checks on the patient’s 6th day to 12th day.

	Sixth day	Seventh day	Eighth day	Ninth day	Tenth day	Eleventh day	Twelfth day
03:00	14.4mmol/L no seizure	19.4mmol/L slight seizure	14.5mmol/L no seizure	12.3mmol/L no seizure	8.5mmol/L no seizure	6.3mmol/L no seizure	4.8mmol/L no seizure
09:00	18.8mmol/L no seizure	21.1mmol/L seizure	17.1mmol/L no seizure	12.0mmol/L no seizure	5.7mmol/L no seizure	4.8mmol/L no seizure	Refuse to measure
15:00	25.1mmol/L seizure	24.6mmol/L seizure	17.1mmol/L no seizure	14.2mmol/L no seizure	8.3mmol/L no seizure	Refuse to measure	discharge
21:00	20.1mmol/L seizure	18.5mmol/L no seizure	18.1mmol/L no seizure	9.0mmol/L no seizure	Refuse to measure	10.5mmol/L no seizure	discharge

On the eighth day in the hospital, we evaluated the patient’s glucose metabolism again, referring to the random blood glucose values of the previous two days, glycosylated hemoglobin(HbA1c)9.9% (normal range 4-6%), fructosamine 2.38 (normal range 1.10-2.14). We asked the endocrinology department to help manage glucose. Patients’ glucose monitoring was changed to every 2 hours, oral antidiabetic medications were discontinued, and Insulin Degludec/Insulin Aspart 18u was administered subcutaneously 5 minutes before breakfast and dinner. The patient’s epileptic seizure did not return and her glucose levels steadily decreased (**Table 1**). On the twelfth day in the hospital, her fructosamine was 2.08. She can be discharged and continued to use Insulin Degludec/Insulin Aspart for a month.

Three months after discharge, we followed up with the patient again in the outpatient department. Without using any drugs, the fasting blood glucose, glycosylated hemoglobin, and blood lipids of the patient were normal without any obvious sequelae.

## 3 Discussion

To the best of our knowledge, this is the first reported case of abruptio placentae and epileptic seizure emerged after the occurrence of perinatal hyperglycaemia in woman with GDM and HTG AP in the second trimester. Some reasons may explain this phenomenon. On the one hand, acute pancreatitis usually occurs in the third trimester (52%), postpartum (30%), and rarely in the second trimester (3–5). Because gestational lipids typically peak in the third trimester of pregnancy, which is determined by estrogen-induced triglyceride synthesis and very low-density lipoprotein (7). In particular, HTG AP accounts for only 5% of cases (8) (**Table 2**). On the other hand, the morbidity of abruptio placentae and seizure decreased with the improvement of prenatal screening and medical care. Although the incidence is decreasing, both are still serious adverse events and can be seriously harmful during pregnancy. According to the literature, abruptio placentae and epileptic seizure are not considered to be common complications of HTG AP. As a result, the current knowledge of clinicians is likely to be insufficient in the event of a bursty abruptio placentae and epileptic seizure in patients with GDM and HTG AP.

**Table 2:** The etiology of Pancreatitis in pregnancy.

Proportion of the etiology		
Gallstones (65–68%)		
Alcohol abuse (5–10%)		
Familial hypertriglyceridemia-induced pancreatitis (5%)		
diopathic (15%)		
Drugs-induced AP (thiazide diuretics) (cases)		
Pancreatitis associated with pregnancy-induced hypertension (cases)		
Acute fatty liver of pregnancy associated with AP (cases)		
Hyperparathyrodism (cases)		
Gene mutations (cases)		
Cationic trypsinogen (PRSS1)		
CFTR		
PSTI		
PPARG		

AP, acute pancreatitis; CFTR, cystic fifibrosis transmembrane conductance regulator; PPARG, peroxisome proliferator-activated receptor gamma; PSTI, pancreatic secretory trypsin inhibitor.

Abruptio placentae is a pregnancy complication that can endanger the life and health of the mother and fetus. Previous literature studies have suggested that the common causes of abruptio placentae include pregnancy-induced hypertension syndrome, severe stress, trauma, improper obstetric care, smoking, etc (9, 10). HTG AP can be considered as severe stress. However, cases of abruptio placentae after HTG AP alone have been extremely rare in previous studies, suggesting that other mechanisms may be involved. Theoretically, hyperglycemia during pregnancy can lead to placental vascular endothelial dysfunction (11–14), hypercoagulable state of the blood system (15, 16), fetal distress (17), etc. It may facilitate the occurrence of abruptio placentae, but the specific mechanism needs to be investigated further. Consistent with the above studies, our patient had HTG AP which occurred with GDM. Next, abruptio placentae did occur after occurrence of perinatal hyperglycemia. Therefore, for pregnant women with HTG AP and GDM, if they have lower abdominal pain, vaginal bleeding, and other suspected manifestations, clinicians should increase the awareness of abruptio placentae, and early diagnosis is important because in severe abruptio placentae, the fetal mortality rate is nearly 100%, and the maternal mortality rate can be up to 5% (18, 19).

Previous studies have suggested that seizure during pregnancy is more common in epilepsy, eclampsia and stroke (20), and the fact that the patient’s previous medical history, blood pressure and cranial CT were normal at the time of the attack essentially ruled out the possibility of the above conditions. In addition, the patient had normal body temperature and serum calcium, which also excluded the possibility of hyperpyretic convulsion and hypocalcemic convulsion. The cause of epileptic seizure in this patient was unknown. In previous reports, uncontrolled hyperglycemia can also cause seizure (21–23), which may be related to its brain damage (23–27). Most cases have been described in patients with non-ketotic hyperglycemia (NKH), which is a common complication of type 2 diabetes (28, 29). Fewer cases have been described in patients with GDM. Taken together with our case, epileptic seizure may occur only in specific states of stress. In agreement with previous findings (30), this patient’s seizure ceased after the hyperglycemia was corrected. Therefore, for those patients, if an unexplained epileptic seizure occurs, rapid recognition of a hyperglycemic state is vital because the hyperglycemia-induced seizure is commonly refractory to anti-epileptic drugs, and some treatments (phenytoin) may even aggravate them.

Based on the above discussion, these two rare complications in this patient do not seem to rule out the effect of hyperglycemia. However, previous studies on the treatment of pregnant women with HTG AP have focused on lipid reduction, as it has been established in numerous studies that lipid levels are positively correlated with the severity of the disease and adverse fetal outcomes (31, 32), and that early lipid reduction can reduce complications and mortality (33). As a result, numerous studies (34, 35) have focused on the design of different lipid-lowering regimens and the comparison of their efficacy that these regimens did achieve excellent results in reducing mortality and critical illness rate. Thus, the importance of glycemic control in reducing complications is overshadowed. Given the association of prolonged glucose load with increased risk of diabetes-related complications and mortality (36, 37), effective early glycemic control is confirmed critical to achieve sustained and long-term reductions in diabetes-related complications and thereby to reduce mortality and cost of diabetes care related to Type 1 diabetes or Type 2 diabetes (38–40). Yet very little is known about perinatal hyperglycemia. Due to a lack of understanding of its rare complications and deleterious effects, glycemic management was initially neglected after she passed the acute stage. Then the patient’s blood glucose went out of control and seizures returned. As a result, hyperglycemia may not be easy to control after the onset of HTG AP and it is critical to give stricter management of glucose for puerperal women with a history of GDM. Insulin therapy in the acute phase is well defined. However, there is no uniform standard for the selection of hypoglycemic agents for puerperal women who have passed the acute phase of HTG AP.

Because most postpartum women have lactation needs, the drug selection is generally the same as for pregnant women. As a result, only a limited number of oral drugs are currently available for clinical use. Metformin, the most studied oral hypoglycemic drug, is labeled as a Class B drug, meaning there is no strong evidence of a contraindication in pregnant women (41).In terms of the actual efficacy of glycemic control, a systematic analysis involving a total of 4533 GDM patients (42)confirmed that compared to insulin, metformin still had a significantly stronger 2h-postprandial blood glucose control (22 studies, 2301 patients, MD, −1.11; 95% CI −1.50 to −0.72; p < 0.00001), lower HbA1c (15 studies, 1370 patients, MD, −1.04; 95% CI −1.47 to −0.61; P<0.00001), lower gestational FBG(32 studies 2996 patients, MD, −0.89; 95% CI−1.19 to−0.58; P<0.00001). This is consistent with several previous meta-analyses showing that metformin is no less effective or even better than insulin in controlling the primary outcome of GDM (43–46). In addition, there is additional evidence of the advantages of metformin such as ease of administration, ease of patient education, better adherence, and lower cost (47–49). Thus, patients may prefer metformin to insulin in clinical practice (50).

Based on these advantages, metformin has been recommended in the latest Chinese guidelines for the treatment of GDM when patients refuse to use insulin, cannot safely inject insulin, or cannot afford the cost of insulin (6). Our patient was in a similar situation and had passed the acute period. Following the guidelines, we tried metformin to lower blood glucose, but there was no significant reduction in glucose. Given the damage caused by pancreatitis, the slow onset time of oral medication, and the short duration of use, this result should be interpreted with caution and cannot be entirely denied for the effect of metformin. In addition, considering that the long-term effects of metformin on neonates through milk secretion have not been completely elucidated, its safety cannot be absolutely guaranteed. For puerperal women with a history of GDM, the use of metformin to control glucose may not be appropriate even if they have passed the acute phase of HTG AP, and it is still necessary to consider the benefits and risks with caution before using metformin.

Insulin is another agent that can be used to lower blood glucose levels in pregnant women. Considering the long-term safety and non-teratogenicity of insulin, the American Diabetes Association (ADA) and the American College of Obstetricians and Gynecologists (ACOG) had recommended insulin as the primary medical treatment for GDM if lifestyle interventions do not meet glycemic treatment goal (51, 52). For women with the acute disease in the perinatal period, the principle of controlling maternal hyperglycemia with insulin has long been recognized, while there remains no nationwide or international consensus about the choice of infusion method and the type of insulin, and most national endocrine and obstetric governing bodies have not published specific guidelines.

In the intrapartum period, the American College of Obstetricians and Gynecologists (ACOG) recommended a continuous insulin infusion to maintain blood glucose levels at rv100 mg/dL using a protocol adapted from Coustan (1, 53). The protocol did not adequately take into account differences in insulin resistance levels among pregnant women. However, various institutions still choose continuous glucose and insulin infusion to manage intrapartum glucose, despite poor evidence for this decision (54). Another protocol, from Northwestern Memorial Hospital’s Prentice Women’s Hospital, involved administration of insulin and dextrose titration by an endocrinologist based on every 2 hours capillary blood glucose. Its medical decisions relied heavily on the clinical experience of numerous specialized endocrinologists, which is obviously cumbersome and inefficient. In 2011, Northwestern University began developing a new protocol for managing glucose, creating standardized algorithms in which registered nurses titrated insulin at different rates based on hourly capillary glucose checks. They also designed a series of tables (**Table 3**) to instruct providers on insulin administration, depending on the patient’s total daily dose of insulin combined with the patient’s cumulative basal and bolus insulin requirements and insulin resistance (55). This protocol was simple to implement and improved the consistency of glucose management. Moreover, it was once tailored to the individual needs of different patients. Our patients who received this regimen in the intensive care unit had excellent glycemic control and no seizures. However, this protocol requires frequent glucose measurements by specialist nurses and its relative complexity and intensiveness when glucose levels may change rapidly, which is difficult to administer in general wards. We need further research to clarify the optimal glucose infusion protocol for patients in general wards.

**Table 3:** The new protocol for managing glucose from Northwestern University.

Table 1: Total Daily Dose of Insulin≤60 Units/24 hours							
Hourly	Initial Dose of Insulin		Continuous infusion	CBG UNCHANGED or INCREASING		CBG DECREASING	
CBG Mg/dL	Bolus Units IV push	Basal Units/hour	D_10_ W ml/hr	Bolus Units IV push	Basal Units/hour	Bolus Units IV push	Basal Units/hour
<70	0	0	50	0	0	0	↓0.5
70-100	0	0	50	0	no△	0	↓0.3
101-130	1	0.5	50	1	↑0.5	0	no△
131-160	2	0.5	50	2	↑0.5	0	↑0.5
161-190	3	0.5	0	3	↑0.7	1	↑0.5
191-220	4	0.5	0	4	↑0.7	2	↑0.8
>220	5	0.5	0	5	↑0.8	3	↑0.8
Table 2 Total Daily Dose of Insulin 61-120 Units/24 hours							
Hourly	Initial Dose of Insulin		Continuous infusion	CBG UNCHANGED or INCREASING		CBG DECREASING	
CBG Mg/dL	Bolus Units IV push	Basal Units/hour	D_10_ W ml/hr	Bolus Units IV push	Basal Units/hour	Bolus Units IV push	Basal Units/hour
<70	0	0	50	0	0	0	↓0.4
70-100	0	0	50	0	no△	0	↓0.4
101-130	2	1.0	50	2	↑0.6	0	no△
131-160	3	1.0	50	3	↑0.6	0	↑0.6
161-190	4	1.0	0	3	↑0.8	2	↑0.6
191-220	5	1.0	0	5	↑0.8	3	↑0.8
>220	6	1.0	0	6	↑1.0	4	↑0.8

If CBG is < 70, give 100mL of D10 W over 10 minutes followed by the 50mL/hr continuous infusion.

↑ It represents an increase in the insulin dose.

no△ It represents insulin dosage does not need to change.

In the postpartum period, the Guideline of Committee on Practice Bulletins—Obstetrics states that women with gestational diabetes discontinue insulin at postpartum stage (56), which is consistent with clinical practice. Therefore, there is relatively little data on the use of insulin in the treatment of postpartum hyperglycemia, especially in patients with combined pancreatitis. In our patient, after the acute phase, she preferred subcutaneous injections of insulin analogue twice daily to continuous subcutaneous insulin infusion. However, the results showed acceptable effects of glucose control. Thus, intermittent injection appears to be an alternative in postpartum hyperglycemia.

Another controversial issue is the type of insulin used. In the current consensus, short-acting and intermediate-acting human insulin are the preferred insulin regimens for GDM (57). However, it is unclear whether this applies to postpartum, and the specific insulin has not been confirmed. Numerous studies of GDM have used Novolin 30R as an object. However, a meta-analysis by Li et al. (42) confirmed that Novolin 30R’s efficacy was even inferior to that of metformin. Additionally, like other premixed insulin, it has the inability to adjust the long- and short-acting components separately or adequately treat post-lunch and early-morning hyperglycemia (58). Finding appropriate insulin is a key issue in current postpartum glucose management. We used Insulin Degludec/Insulin Aspart(IDegAsp) in our case. IDegAsp is the first fixed-ratio co-formulation of insulin degludec, which provides long-lasting basal insulin coverage, and insulin aspart, which targets postprandial glucose (59). It has the advantages of rapid onset, longer half-life, flat and stable glucose lowering profile, less 24-H variability, and lower risk of hypoglycemia (60). As a result, it has fewer injections and is more acceptable to patients. Many high-quality meta-analyses have confirmed its positive glucose lowering effects in type 2 diabetes. However, little is known about its use in postpartum hyperglycemia. Our case provides a valuable reference for its application to postpartum hyperglycemia. However, the long-acting component”degludec insulin” is not approved and is a category C agent in pregnancy yet. Given the potential risks, this recommendation may only be appropriate for those who do not need to breastfeed postpartum.

It must be admitted that there are some limitations in this study. First, with only one case reported in this study, there is relatively limited evidence-based evidence to support its conclusions, which limits its generalizability. Second, there were confounding factors in the study, such as irregular prenatal check-ups, unclear maternal pregnancy status and fetal intrauterine development, lack of pre-onset glucose monitoring, and no confirmation of seizure by electroencephalogram. All of these factors may affect the interpretation of the results. Finally, there are no published randomized controlled trials of IDegAsp in pregnant women, the pregnancy safety of IDegAsp is not sufficiently established, which may inherently limit its clinical applicability in pregnant women. Thus, the conclusions still require further careful interpretation and clinical identification.

## 4 Conclusion

The harms of perinatal hyperglycemia are still not fully understood and can be exacerbated by co-morbidities such as HTG AP and GDM. However, as serious and rare complications can be triggered, effective glucose management is extremely critical. For perinatal women, timely adjustment of continuous insulin infusion according to blood glucose monitoring seems to be the optimal plan, but for women who have survived the acute phase of the disease or be in general wards, our case supports that intermittent subcutaneous injection of a fixed-ratio co-formulation of insulin analogues (such as IDegAsp)may be a suitable alternative. More research is needed to clarify the management of perinatal hyperglycemia in both acute and chronic conditions.

## Data availability statement

The original contributions presented in the study are included in the article/supplementary material. Further inquiries can be directed to the corresponding author.

## Ethics statement

Written informed consent was obtained from the individual(s) for the publication of any potentially identifiable images or data included in this article. Written informed consent was obtained from the participant/patient(s) for the publication of this case report.

## Author contributions

YH and JC conceived and designed the study. ZH and CW collected the data. YH and JC drafted the manuscript. All authors read, edited, and approved the final manuscript. All authors had full access to all the data in the study and take responsibility for the integrity of the data and the accuracy of the data analysis. JC is responsible for the overall content of the manuscript, and serves as the guarantor.
