# Corrigendum: Case report: Abruptio placentae and epileptic seizure after occurrence of perinatal hyperglycaemia in woman with gestational diabetes mellitus and hypertriglyceridemia-induced acute pancreatitis

**DOI:** 10.3389/fendo.2023.1343681

**Published:** 2023-12-14

**Authors:** Yanlang He, Zhijie Huang, Changli Wei, Jianyong Chen

**Affiliations:** ^1^ Medical College of Nanchang University, Nanchang, China; ^2^ Department of Gastroenterology and Hepatology, Jiangxi Provincial People’s Hospital, The First Affiliated Hospital of Nanchang Medical College, Nanchang, China

**Keywords:** abruptio placentae, epileptic seizure, hyperglycaemia, hypertriglyceridemia-induced, gestational diabetes mellitus

In the published article, there was an error regarding the affiliations for Yanlang He. As well as having affiliation 1, they should also have affiliation 2, *Department of Gastroenterology and Hepatology, Jiangxi Provincial People’s Hospital, The First Affiliated Hospital of Nanchang Medical College, Nanchang, China*.

The authors apologize for this error and state that this does not change the scientific conclusions of the article in any way. The original article has been updated.

